# Effectiveness of the ‘Girls Active’ school-based physical activity programme: A cluster randomised controlled trial

**DOI:** 10.1186/s12966-018-0664-6

**Published:** 2018-04-25

**Authors:** Deirdre M. Harrington, Melanie J. Davies, Danielle H. Bodicoat, Joanna M. Charles, Yogini V. Chudasama, Trish Gorely, Kamlesh Khunti, Tatiana Plekhanova, Alex V. Rowlands, Lauren B. Sherar, Rhiannon Tudor Edwards, Thomas Yates, Charlotte L. Edwardson

**Affiliations:** 1 Diabetes Research Centre, University of Leicester, Leicester, UK; 2 NIHR Leicester Biomedical Research Centre, Leicester, UK; 3 Leicester Diabetes Centre, University Hospitals of Leicester, Leicester, UK; 4 Centre for Health Economics and Medicines Evaluation, Bangor University, Bangor, UK; 5 Department of Nursing, School of Health, Social Care and Life Sciences, University of the Highlands and Islands, Inverness, UK; 6 http://www.clahrc-em.nihr.ac.uk/; 7 Sansom Institute for Health Research, University of South Australia, Adelaide, Australia; 8 National Centre for Sport and Exercise Medicine, School of Sport, Exercise and Health Sciences, Loughborough University, Loughborough, UK

**Keywords:** Intervention, Physical education, Teacher, Adolescent female

## Abstract

**Background:**

Globally, adolescent girls’ physical activity (PA) levels are low. The ‘Girls Active’ secondary school-based programme, developed by the Youth Sport Trust, aims to increase PA in adolescent girls. This paper explores the effectiveness of the ‘Girls Active’ school-based PA programme.

**Methods:**

A random sample of girls aged 11–14 from 20 secondary schools (Midlands, UK) participated in a two-arm cluster randomised controlled trial. Ten schools received Girls Active and 10 continued with usual practice. Measurements were taken at baseline, seven- and 14-month follow-up. Primary outcome: wrist-worn accelerometer measured moderate- to vigorous-intensity PA (MVPA). Secondary outcomes: overall PA, light PA, sedentary time, body composition, and psychosocial outcomes. Generalised estimating equations, adjusted for school cluster and potential confounders, were used and A priori subgroup analysis was undertaken. Micro-costing and cost-consequence analyses were conducted using bespoke collection methods on programme delivery information. Outcomes for the cost-consequence analysis were health related quality of life measured by the Child Health Utility-9D and service use.

**Results:**

Overall, 1752 pupils participated, 1211 (69.1%) provided valid 14-month accelerometer data. No difference in MVPA (mins/day; 95% confidence intervals) was found at 14 months (1.7; -0.8 to 4.3), there was at seven months (2.4; 0.1 to 4.7). Subgroup analyses showed significant intervention effects on 14-month in larger schools (3.9; 1.39 to 6.09) and in White Europeans (3.1; 0.60 to 6.02) and in early maturers (5.1; 1.69 to 8.48) at seven months. The control group did better in smaller schools at 14-months (-4.38; -7.34 to -1.41). Significant group differences were found in 14-month identified motivation (-0.09; -0.18 to -0.01) and at seven months in: overall PA (1.39 m*g*/day; 0.1 to 2.2), after-school sedentary time (-4.7; -8.9 to -0.6), whole day (5.7; 1.0 to 10.5) and school day (4.5; 0.25 to 8.75) light PA, self-esteem. Small, statistically significant, differences in some psychosocial variables favoured control schools. Micro-costing demonstrated that delivering the programme resulted in a range of time and financial costs at each school. Cost-consequence analysis demonstrated no effect of the programme for health related quality of life or service use.

**Conclusions:**

Compared with usual practice, ‘Girls Active’ did not affect 14-month MVPA.

**Trial registration:**

ISRCTN10688342

## Background

Youth physical activity (PA) levels worldwide [1] and in the UK [2] are low despite the physiological and psychological benefits [3]. Adolescent females have been highlighted as a particular group at risk of declining PA with recent data showing that only 16% and 9% of girls aged 11–12 and 13–15 years, respectively, are sufficiently active [2]. Although the decline in PA may begin prior to the transition between primary and secondary (high) school, and occurs in both boys and girls [4], there is a need to identify ways to stem the decline in PA in girls once they enter secondary schools as the decline is steeper and faster in girls than boys. Schools have been highlighted as a setting to tackle the inactivity crisis [5–7] using a variety of strategies [8–12]. Effects are generally small and short-term [11, 13]. Furthermore, potential mediators of physical activity changes (i.e. to help explain effectiveness) are not robustly or routinely assessed [14]. Reviews have identified that much of the evidence comes from the US, [8, 9] and there is an absence of robust “proven” or “promising” practice within the UK [15].

In an effort to tackle youth inactivity the Youth Sport Trust (YST; the largest non-profit organisation focussing on youth sport and activity in the UK) have developed programmes designed to support schools in engaging girls in PA. ‘Girls Active’ is one such programme which uses peer leadership and marketing to empower adolescent girls to influence school decisions, develop themselves as role models, and promote PA to peers. Schools are provided with training and resources to review the their PA, sport and physical education (PE) provision, culture and practices to girls and to create action plans for how they will implement changes. The programme had not been robustly or independently evaluated.

The aim of this study was to assess the effectiveness of the Girls Active PA programme in UK secondary schools.

## Methods

Ethics approval and study sponsorship were obtained from the University of Leicester. The sponsor had no role in the design, undertaking or reporting of the study. School principals provided written consent for their school to participate. Parents/guardians were provided with an opt out consent form and only pupils who did not return the opt out consent form participated. Participants provided verbal assent prior to each measurement session and could withdraw at any time.

### Design

This cluster RCT had follow-up at seven and 14 months. Following baseline measurements, schools were randomised by an independent statistician to one of two groups stratified by school size (pupil median: < 850, ≥850) and percent black and minority ethnicity (BME) pupils (median: < 20%, ≥20%). Sequentially numbered sections within a folder were used to implement the group allocations. The investigator team were not aware of the sequence until after randomisation. Measurement team members, except the team lead for the day, were blinded to group randomisation. The trial statistician was not blinded. However, the statistical analysis plan was signed off prior to database lock and any deviations from the analysis plan are reported herein.

### Stakeholder involvement

Girls Active is built on over 10 years of work by the YST that includes consultations with school senior leaders, teachers, young people, national agencies and corporate partners. This extended to consultations with adolescent girls and their teachers in order to refine the programme prior to this evaluation study. The research team consulted with two groups (*n* = 8 and *n* = 6) of adolescents and PE teachers (*n* = 3) at local schools in Leicester City ahead of the funding application. This informed the decision around outcome measures: i.e. preference of a wrist worn accelerometer over one worn on the hip or the thigh, economic evaluation cost diaries and process evaluation themes and questions. Key stakeholders were involved in the study as two lay members sat on the trial steering committee (TSC). Lead teachers co-designed their own school reports so that anonymised baseline data specific to their school could be used within their schools. Further dissemination will be undertaken to the study participant through a further anonymised school level report as well as a briefing event for school leads and other personnel involved in education, sport and physical activity decision making. Stakeholders were not involved in decisions around study design, recruitment or conduct of the study and the burden of the programme was not assessed.

### Participants and school clusters

All state (government funded) secondary schools in Leicester City, Leicestershire and Rutland (LLR), UK (*n* = 56) with a Key Stage 3 (KS3: age 11–14 years) were eligible and were sent a letter of invitation to the study. Of these, 25 agreed to come to a briefing event about the study, 15 attended of which 14 obtained consent from the school principal for their school to participate. In tandem, a further 26 state schools that were geographically close to LLR but in neighbouring counties were approached and 6 of these consented to participate. Schools provided the research team a list of all eligible girls between the ages of 11 and 14 years and in years 7, 8 and 9. All eligible pupils were provided with an information pack that contained a separate participant and parent/guardian information sheet and opt out consent form as well as an invitation letter. Pupils had two weeks to return the opt out consent form. Using a random number generator, 90 girls from each school were chosen at random (split between year groups).

### Sample size

This study was designed to provide adequate power to detect a meaningful group difference [16] in MVPA of 10 mins/day assuming a standard deviation of 18 mins/day in MVPA [17], a power of 90%, a significance of 0.05, a cluster size of 56 girls and an intra-class correlation of 0.1. Twenty schools and ≥ 80 girls/cluster allowed for cluster attrition and 30% loss to follow-up and non-compliance with accelerometer wear.

### Participant assessments

Measurement sessions took place at school during the school day. There was an explanation of methods, the assent process, question and answers and the accelerometer protocol.

#### Objective PA

Methods used in this study have been described in the protocol paper [18]. Briefly, girls wore a GENEActiv accelerometer 24 h/day for seven days on their non-dominant wrist at all time-points and were given a £5 gift voucher on return of the device with at least 4 days of data collected. The GENEActiv devices were initialised with a sampling frequency of 100 Hz and set to start recording at midnight on the first day of data collection and stop recording at midnight seven days later.

#### Data processing

GENEActiv .bin files were analysed with R-package GGIR v1.2–11 (http://cran.r-project.org) [19, 20]. Variables of interest were calculated over the 24 h day using published thresholds: MVPA, overall PA (average acceleration; derived using the Euclidean Norm Minus One method), light PA and sedentary time [21, 22]. Sedentary time was partitioned out from sleep using the nocturnal sleep detection algorithm in GGIR [23]. The meeting of PA guidelines [24] was calculated as those achieving ≥ 60 mins MVPA on each measurement day. Accelerometer variables were calculated for the whole day and by specific periods: during school, after school (up to 9 pm), school days and non-school days. Each school’s own start and end times were used to define school hours variables. PA data analysis include participants with ≥ 16 h of wear-time during each 24 h period [25] on ≥ 2 days including ≥ 1 school day.

#### Psychological outcomes

A range of psychological factors that may mediate PA participation were self-reported on a paper-based questionnaire as described in the protocol paper [18]: intentions and motivation to be active; attitudes to PA; perceived family, peer and teacher social support for PA; perceptions of the school social and physical environment; PA self-efficacy and enjoyment; perceived importance of PA, and physical self-perceptions (self-esteem, body attractiveness and physical self-worth).

#### Anthropometric and body composition measurements

Standing and sitting heights (Seca 213 stadiometer, Seca, Birmingham, UK) and weight (Tanita SC-330ST, Tanita Europe BV, Middlesex, UK) were assessed to the nearest 0.1 cm and 0.1 kg, respectively, using standardised procedures. Body mass index (BMI) was calculated and converted into z-scores relevant to the UK population [26]. Percentage body fat was estimated using pediatric scales (Tanita SC-330ST, Tanita Europe BV, Middlesex, UK).

#### Potential covariates

Participant age (in months) was calculated from date of birth, year group was self-reported, and socioeconomic status was represented by calculating the index of multiple deprivation (IMD) from participants’ self-reported postcode. Age at peak height velocity (APHV) was used as an indicator of biological maturity category [27].

### School level data

School level socioeconomic position/deprivation was represented by the percentage of pupils eligible for free school meals (%FSME) from the relevant 2015 school census [28]. School pupil numbers were reported by the lead teacher and verified by 2015 school census data.

### Intervention group – Girls Active programme

**Fig. 1:**
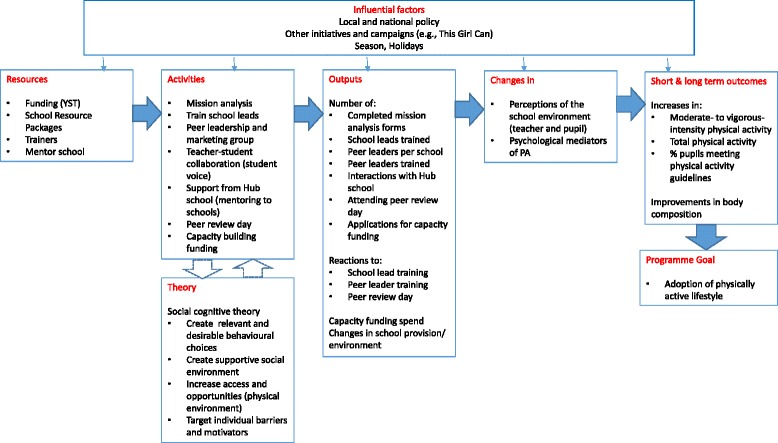
Proposed logic model for the Girls Active programme

Details of the Girls Active programme have been described in the protocol paper [18]. The aim of Girls Active is to provide a support framework for schools to review and change their PA, PE and school sport culture and practices with the support of the YST and a hub school. Teachers completed a school self-review and attended an initial training day delivered by a YST national tutor. At this day, teachers discussed the programme elements (including establishing a peer leader group) and started developing their school action plan. They received a folder of case studies and electronic versions of marketing materials for promotion within their school. One of the key elements to the programme was the formation of a girls leadership and peer marketing group to empower girls to influence PE, sport and PA in their school, develop as role models, and promote and market PA to other girls. Over the course of the programme, resources for lead teachers were uploaded to an online file sharing system. School leads attended a peer review day to share practice with other teachers facilitated by the development coach and a hub school teacher. Lead teachers were offered in-person or phone support through the hub school or development coach. Lead teachers were free to implement the programme flexibly in whatever way they wished but were encouraged to set up a peer leader group who were to market PA to their peers and help prioritise PA decisions within their school. Teachers were encouraged to identify pupils that are not necessarily the sporty pupils but those who bring a range of perspectives and could communicate with, and motivate their peers. They were provided with two £500 capacity funding instalments to coincide with action plan submission. The programme was delivered in the same manner as would have been done in the real-world setting and, in theory, Girls Active has the potential to continue on in schools if implemented with sustainability and embedding in mind. The elements of the “off-the-shelf” programme which was originally developed without specific reference to theory were mapped to constructs in social cognitive theory post-hoc by the academic team (Fig. 1). Seven core components of Girls Active were identified: submission of the first self-review and action plans; attendance of lead teacher at initial training; use of package of resources or use of an alternative; engagement of young people as peer leaders; use of online, in person or phone support of hub and/or development coach; lead teacher attendance at peer review day; and submission of the second mission analysis. The intervention was delivered at the cluster level but the participant data reported herein were collected at the individual level.

### Control group - usual practice

Control arm schools were not given any specific guidance or advice and were assumed to carry on with their usual practice of PE and sport provision.

### Primary and secondary outcomes

The primary outcome was difference in the change in mean mins/day of MVPA at 14-month follow-up between groups. Secondary outcomes included: overall PA; sedentary time; and all PA outcomes split by weekday and weekend day, and during school hours and after school hours; BMI z-score; percentage body fat; and psychological factors that may mediate PA participation as described above.

### Economic analysis

A full description of the economic assessment methods of the Girls Active trial are available [29]. Briefly, micro-costing methodology [30] was applied to calculate the costs of delivering the programme over a whole school year for the intervention arm schools. This provides a mean cost per school. Money and time spent on delivering Girls Active were self-reported by lead teachers using two types of bespoke cost diaries and a questionnaire and, if needed, these were followed up with a phone call for clarification. The cost-consequence analysis was conducted from a public sector, multi-agency perspective. Health related quality of life measured by the Child Health Utility-9D [31] was used as the measure of effect. Use of GP and school based services (school nurse and school counsellor) were used as the measure of costs. These were all self-reported by participants at each timepoint.

### Statistical analysis

The results are reported according to the CONSORT statement for cluster RCTs [32]. Primary and secondary outcome analyses were based on a complete case analysis. Intention to treat (ITT; all schools and recruited pupils were analysed in the group they were randomised to) and per protocol analyses were also undertaken for the primary outcome as sensitivity analyses. The per protocol population included schools that engaged with 70% of the seven core components (as detailed above) of the programme over the 14 months and had complete data for the analysis concerned on ‘by analysis’ basis. In the control arm, the per protocol population included all schools/pupils randomised to that arm.

Generalised estimating equations, accounting for school level clustering, and adjusting for baseline MVPA, stratification factors of school size (< 850, ≥ 850) and % of non-White pupils (< 20%, ≥ 20%), %FSME and participant year group, were employed. Subgroup analyses involved within subgroup stratification and between subgroup interactions effects between the intervention arm and pre-specified subgroups: baseline school (social deprivation and size) and pupil (ethnicity, maturation status, year group, PA level) characteristics. Sensitivity analyses explored the effect of the number of valid accelerometer days and the season of data collection. All analyses were performed using Stata (v.14.0), with statistical significance set as *p* < 0.05. Changes from the agreed statistical analysis plan included the addition of %FSME (school SES) and participant year group as covariates in the primary and secondary outcomes analyses.

## Results

**Fig. 2:**
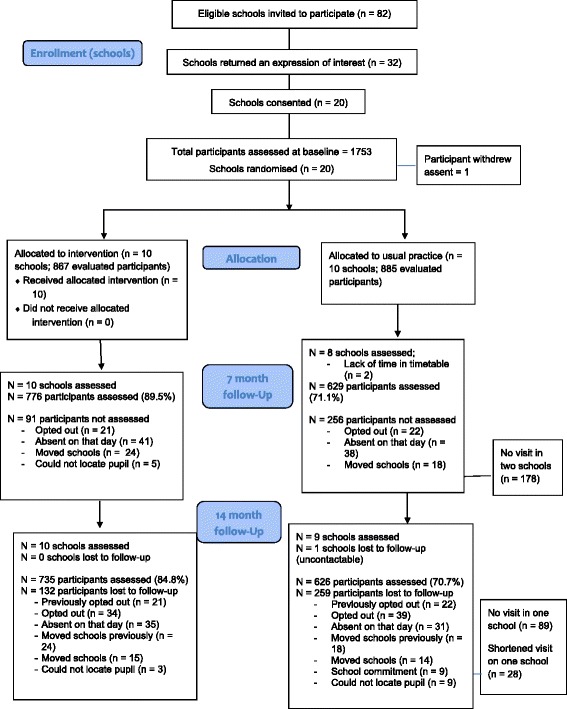
CONSORT flow chart for the Girls Active cluster randomised controlled trial

In Spring 2015, 20 secondary schools were recruited (Fig. 2). Of these, 18 schools agreed to be followed-up at seven months, and 19 at 14 months. From these schools, 1752 adolescent girls provided assent and participated at baseline (Feb – April 2015), 1405 (80.2%) at seven months (Sept – Nov 2015), and 1361 (77.7%) at 14 months (April – June 2016). Tables 1 and 2 present the baseline characteristics of the schools and participants, respectively. Participants who did not complete the 14 month assessment (*n* = 301) were older (*p* < 0.001), had a higher BMI z-score (*p* = 0.021) and provided 0.2 days less accelerometer data (*p* < 0.001) at baseline (Table 3). Complete accelerometer data (i.e., ≥ 2 valid days, including at least one school day at baseline and 14 months) was available for 1211 participants (69.1%) for the primary outcome analysis at 14-month follow-up. No serious adverse events/reactions were reported.

**Table 1:** Baseline characteristics of each school (cluster) by randomised group; usual practice (control) or the Girls Active programme (intervention)

Characteristics	Control (*n* = 10)	Intervention (*n* = 10)	Total (*n* = 20)
Cluster Level			
Number of participants, n (SD)	89 (2.0)	87 (3.0)	88 (2.0)
Non-White participants, % (SD)	21.3 (27.7)	19.3 (24.1)	20.3 (25.3)
Overall School Characteristics			
Full-time pupils, *n* (SD)	977 (199)	859 (386)	918 (305)
Non-White pupils, % (SD)	25.4 (34.2)	21.6 (28.0)	23.5 (30.5)
FSME, % (SD)	9.7 (6.2)	13.3 (5.6)	11.5 (6.1)
IMD decile score, score (SD)^a^	7.2 (2.3)	6.1 (2.5)	6.7 (2.4)
IMD rank score, score (SD)	21,840 (8013)	18,949 (7860)	20,395 (7866)
Schools ≥ 850 pupils, n (%)	8 (80.0)	6 (60.0)	14 (70.0)

All values, unless otherwise stated, are presented as a mean and standard deviation (SD) across schools; Values are presented as means (standard deviation) for continuous variables and number (%) for categorical variables. *FSME* Free School Meal Eligibility; *IMD* Index of Multiple Deprivation

^a^IMD 2015 decile score ranges 1–10, where 1 is the least deprived and 10 the most deprived

**Table 2:** Baseline characteristics at the individual participant level by randomised group; usual practice (control) or the Girls Active programme (intervention)

Individual level characteristics	Control (*n* = 885)	Intervention (*n* = 867)	Total (*n* = 1752)
Age, years (SD)	12.8 (0.8)	12.8 (0.8)	12.8 (0.8)
Year group categories, *n* (%)			
Year 7	356 (40.2)	318 (36.7)	674 (38.5)
Year 8	355 (40.1)	365 (42.1)	720 (41.1)
Year 9	174 (19.7)	184 (21.2)	358 (20.4)
Ethnicity categories, *n* (%)			
White European	669 (75.9)	673 (77.1)	1342 (76.8)
South Asian	123 (14.0)	81 (9.4)	204 (11.7)
Other	90 (10.2)	112 (12.9)	202 (11.6)
IMD decile, score (SD)^a^	6.5 (2.8)	5.1 (2.7)	5.8 (2.8)
IMD rank, score (SD)	19,649 (9395)	15,322 (8840)	17,505.8 (9375)
Biometric Measurements			
Average standing height, cm (SD)	156.1 (7.9)	155.8 (7.8)	156.0 (7.9)
Average sitting height, cm (SD)	81.5 (4.6)	81.1 (4.6)	81.3 (4.6)
Body mass, kg (SD)	48.7 (12.4)	48.9 (12.4)	48.8 (12.4)
BMI, z-score (SD)	0.14 (1.3)	0.22 (1.3)	0.18 (1.3)
Percent body fat, % (SD)	23.9 (7.6)	24.2 (7.8)	24.1 (7.7)
APHV, years (SD)	12.1 (0.5)	12.1 (0.5)	12.1 (0.5)
BMI categories, *n* (%)			
Underweight	136 (15.7)	129 (15.2)	265 (15.4)
Normal weight	543 (62.6)	530 (62.4)	1073 (62.5)
Overweight	154 (17.7)	149 (17.5)	303 (17.6)
Obese	35 (4.0)	42 (4.9)	77 (4.5)
Biological maturity categories, *n* (%)			
Early	139 (16.4)	125 (15.0)	264 (15.7)
Average	576 (68.0)	570 (68.5)	1146 (68.3)
Late	132 (15.6)	137 (16.5)	269 (16.0)
Accelerometer variables			
MVPA, mins/day [IRQ]	43.9 [30.6-58.0]	41.8 [29.2-56.1]	42.6 [29.9-57.0]
Light PA, mins/day [IRQ]	279.5 [247.8-307.0]	272.5 [244.5-302.7]	275.8 [246.1-305.0]
Sedentary, mins/day [IRQ]	549.4 [508.5-592.1]	552.6 [516.0-594.3]	550.6 [511.7-593.5]
Overall PA, *mg* (SD)	36.7 (8.9)	35.7 (8.8)	36.2 (8.9)
Valid days, number (SD)	6.6 (1.1)	6.8 (0.9)	6.7 (1.0)
Pupil’s achieving ≥ 60 mins MVPA on every valid day, *n* (%)	20 (2.3)	20 (2.4)	40 (2.3)

Values are presented as means (standard deviation) or median [interquartile range] for continuous variables and number (%) for categorical variables

^a^IMD 2015 decile score ranges 1–10, where 1 is the least deprived and 10 the most deprived

*IMD* index of multiple deprivation; *BMI* body mass index; *APHV* age at peak height velocity; *MVPA* moderate- to vigorous-intensity physical activity; *PA* physical activity

Missing data: 2 age; 4 ethnicity; 123 pupils social deprivation score; 30 standing height; 50 sitting height; 7 body weight; 34 BMI; 33 percentage body fat; 73 APHV, biological maturity; 44 MVPA, light PA; 46 pupils meeting PA guidelines, number of valid days; 58 sedentary; 0 all other variables

**Table 3:** Baseline characteristics of completers versus non-completers at 14 month follow-up

Characteristics	Completers (*n* = 1361)	Non-completers (*n* = 301)	*P*-value^a^
Age, years (SD)	12.8 (0.8)	13.1 (0.8)	< 0.001
Year group categories, *n* (%)			
Year 7	571 (41.9)	73 (24.2)	
Year 8	544 (39.9)	147 (48.8)	
Year 9	247 (18.1)	81 (26.9)	< 0.001
Ethnicity categories, *n* (%)			
White European	1014 (74.6)	244 (81.1)	
South Asian	175 (12.9)	28 (9.3)	
Other	170 (12.5)	29 (9.6)	0.060
IMD decile, score (SD)^b^	5.8 (2.8)	5.8 (3.0)	0.678
Biometric measurements			
Average standing height, cm (SD)	155.6 (7.9)	157.8 (7.0)	< 0.001
Average sitting height, cm (SD)	81.1 (4.6)	82.2 (4.1)	< 0.001
Body mass, kg (SD)	48.1 (12.2)	51.3 (12.7)	< 0.001
BMI, z-score (SD)	0.13 (1.3)	0.33 (1.3)	0.021
APHV, years (SD)	12.1 (0.5)	12.1 (0.5)	0.600
Accelerometer variables			
MVPA, mins/day [IRQ]	42.5 [30.2-55.6]	40.8 [30.1-60.2]	0.598
Light PA, mins/day [IRQ]	275 [246-305]	274.9 [246-304]	0.634
Sedentary, mins/day [IRQ]	550 [511-593]	550.7 [514-595]	0.600
Valid days, number (SD)	6.7 (0.9)	6.5 (1.2)	< 0.001
Pupils achieving ≥60 mins MVPA on every valid day, *n* (%)	32 (2.4)	5 (2.0)	0.597

*IMD* index of multiple deprivation; *BMI* body mass index; *APHV* age at peak height velocity; *MVPA* moderate- to vigorous-intensity physical activity; *PA* physical activity

Missing data: 2 age; 2 ethnicity; 110 pupils social deprivation score; 29 standing height; 50 sitting height; 6 body weight; 33 BMI, BMI categories; 56 percentage body fat; 72 APHV, biological maturity; 41 light PA, 43 pupils meeting PA guidelines, 41 number of valid days; 52 sedentary; 0 all other variables

NOTE: Completers included all those who assented at the 14 month visit from all 18 completing schools and the one school with the modified 14 month visit. Non-completers included all those who did not provide assent at the 14 month visit from all 18 completing schools and the one school with the modified 14 month visit. Participants from one school lost to follow-up were not included in either group

^a^*P*-values test for the difference between completers and non-completers and were estimated using either two sample t-test, Chi-squared test or Wilcoxon rank sum test, as appropriate

^b^IMD 2015 decile score ranges 1–10, where 1 is the least deprived and 10 the most deprived

### Primary outcome analysis

There were no differences between intervention and control groups at 14 months for the change in mean mins/day of MVPA in the complete case (1.8 mins/day; 95% C.I. -0.8 to 4.3; *p* = 0.178), intention-to-treat (1.7 mins/day; 95% C.I. -0.6 to 3.9; *p* = 0.158) or per protocol (1.7 mins/day; 95% C.I. -1.15 to 4.48; *p* = 0.246) analyses (Table 4). At seven months, a difference in MVPA between groups was found in the complete case (2.4 mins/day; 95% C.I 0.1 to 4.7; *p* = 0.039), intention-to-treat (2.3 mins/day; 95% C.I. 0.2 to 4.3; *p* = 0.028) and per-protocol (3.1 mins/day; 95% C.I. 0.93 to 5.35; *p* = 0.005) analyses.

**Table 4:** Changes in minutes per day of MVPA at 7 and 14 (primary outcome) month follow-up between participants randomised to usual practice (control) or to the Girls Active programme (intervention)^a^

	Number of schools | pupils		Mean change from baseline (95% C.I.)		Adjusted difference at follow-up^b^		
	Control	Intervention	Control	Intervention	Coefficient (95% C.I.)	*P*-value	ICC
Complete case^c^							
7 months	8 | 572	10 | 730	-6.18 (-7.30 to -5.06)	-2.45 (-3.50 to -1.41)	2.42 (0.13 to 4.72)	0.039	0.03
14 months	9 | 539	10 | 672	-3.47 (-4.91 to -2.03)	-2.09 (-3.27 to -0.90)	1.75 (-0.80 to 4.29)	0.178	0.02
Per protocol^d^							
7 months	8 | 572	8 | 586	-6.18 (-7.30 to -5.06)	-1.91 (-3.11 to -0.72)	3.14 (0.93 to 5.35)	0.005	0.02
14 months	9 | 539	8 | 549	-3.47 (-4.91 to -2.03)	-1.46 (-2.74 to -0.18)	1.67 (-1.15 to 4.48)	0.246	0.02
Intention-to-treat^e^							
7 months	10 | 885	10 | 867	-5.92 (-7.05 to -4.78)	-2.50 (-3.57 to -1.44)	2.30 (0.25 to 4.35)	0.028	-
14 months	10 | 885	10 | 867	-3.63 (-5.03 to -2.23)	-2.01 (-3.20 to -0.81)	1.65 (-0.64 to 3.94)	0.158	-

*CI* confidence interval; *ICC* intra-class correlation; *MVPA* moderate- to vigorous-intensity physical activity

^a^Including pupils who have worn the accelerometer with a minimum of two valid days with at least one school day at baseline and 7 months, and at baseline and 14 months

^b^Difference in the mean MVPA at follow-up adjusted for cluster effect, baseline MVPA value, participant year group, school percentage free school meal eligibility and stratification categories (school size and percentage of non-White pupils)

^c^Those with missing outcome data or missing variables required for the model adjustment are excluded

^d^Schools that did not engage with 70% of the programme have been excluded from this analysis

^e^Missing data imputed using multiple imputation

### Subgroup analysis

**Fig. 3:**
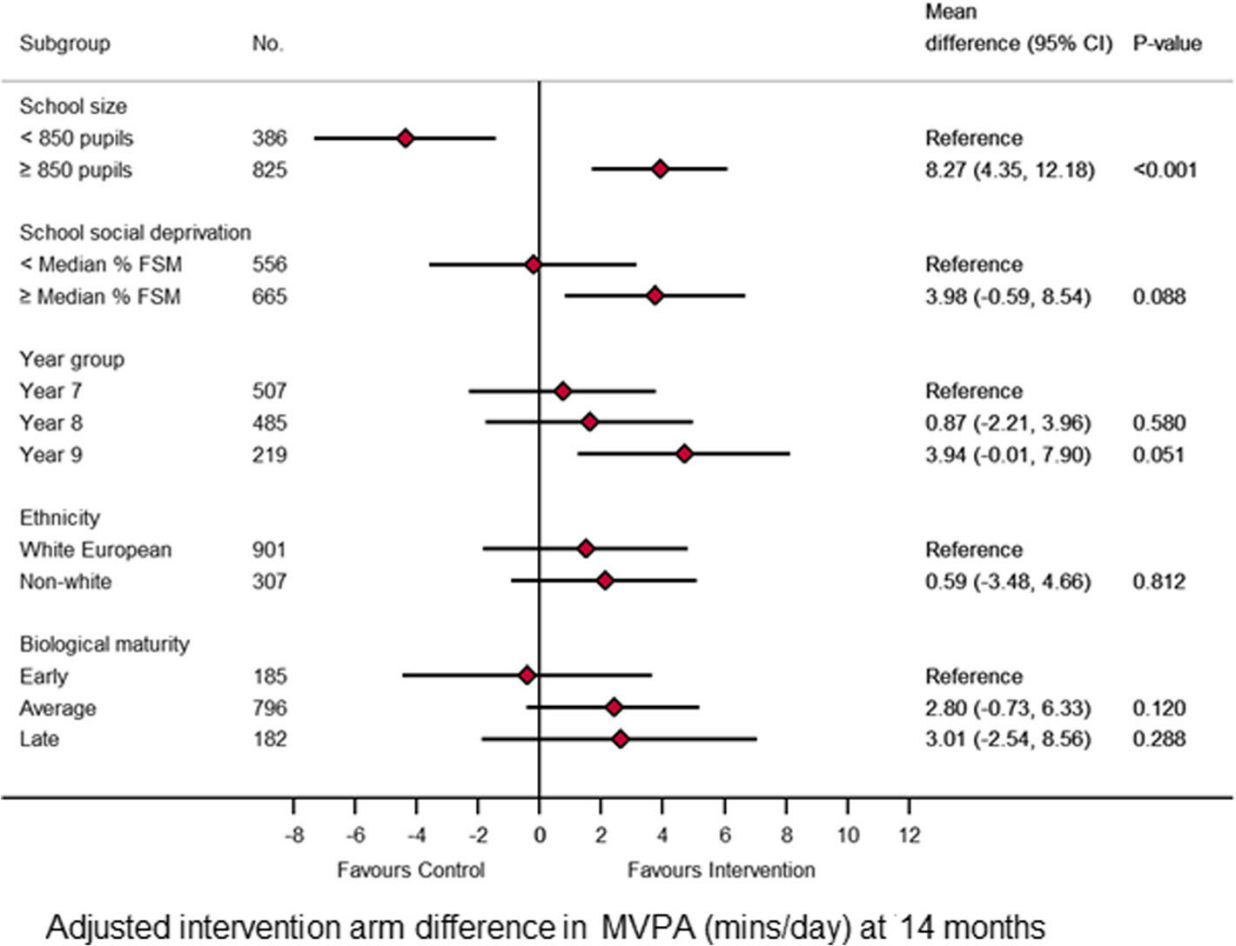
Forest plot of the effect of the intervention at 7 months on the primary endpoint by subgroups

**Fig. 4:**
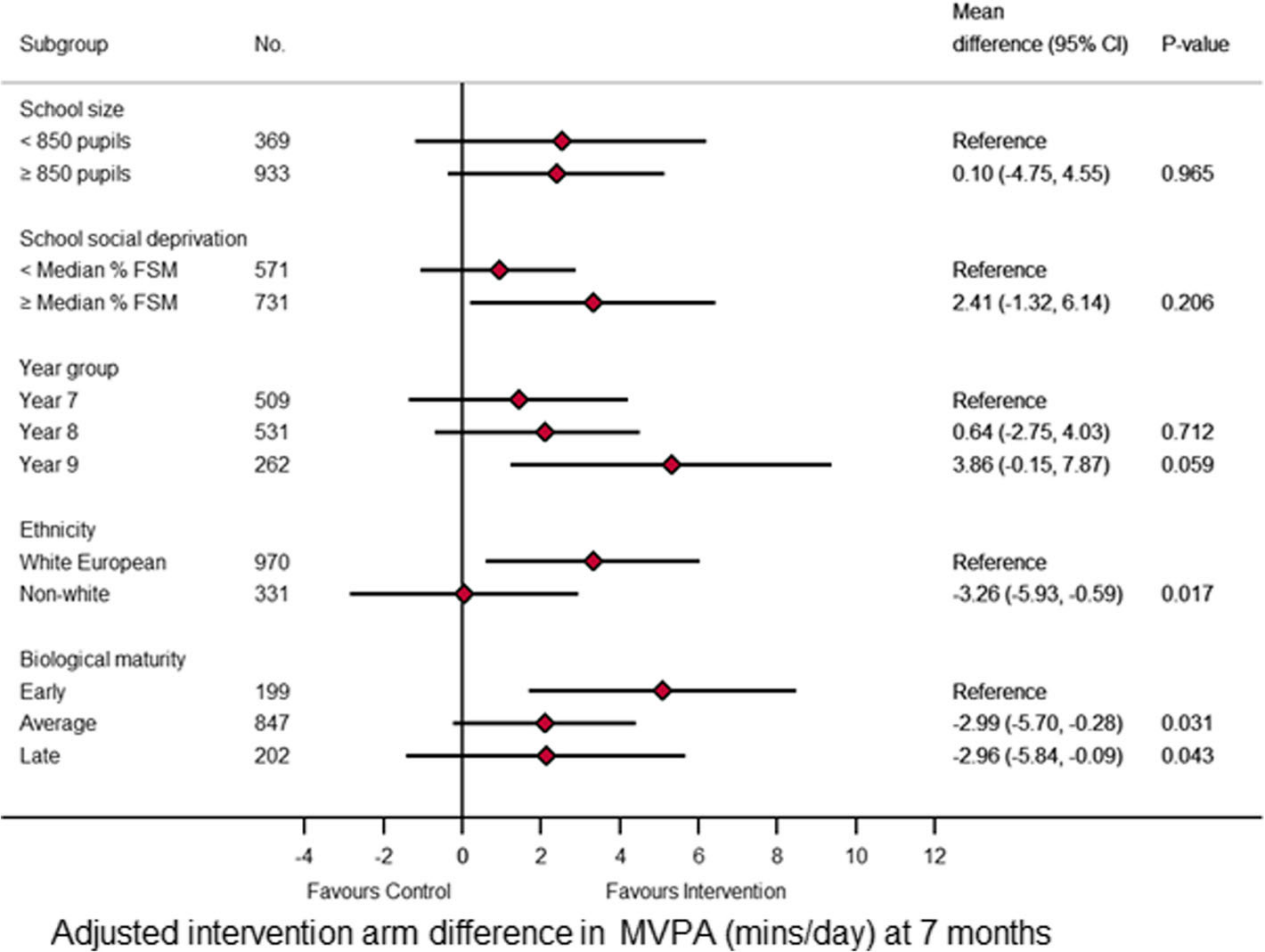
Forest plot of the effect of the intervention at 14 months on the primary endpoint by subgroup

Between subgroup interaction effects revealed subgroup effects (i.e. the programme had a differing effect depending on certain baseline characteristics) at 14 and seven months (Figs. 3 and 4, respectively). At 14 months, the between subgroup interaction effects (*p* < 0.001) revealed a difference between randomised arms of 3.9 mins/day (95% C.I. 1.39 to 6.09; *p* < 0.001) favouring the intervention arm in larger schools (≥ 850 pupils). In smaller schools (< 850 pupils) there was a difference between randomised arms of -4.4 mins/day (95% C.I. -7.34 to -1.41; *p* = 0.004) favouring the control arm. At seven months, in White European and early maturers there was a difference between randomised arms of 3.1 mins/day (95% C.I. 0.60 to 6.02; *p* = 0.017) and 5.1 mins/day (95% C.I. 1.69 to 8.48; *p* = 0.003), respectively, favouring the intervention arm.

### Accelerometer secondary outcomes

No differences were found in the MVPA variables at seven or 14 months when stratified by weekday/weekend or time of day (Table 5). Generally, there were no changes in the other accelerometer variables at seven or 14 months except a significant difference in total PA at seven months (1.4 m*g*; 95% C.I. 0.1 to 2.2; *p* = 0.030), in sedentary time during the after school period at seven months (-4.7 mins/day; 95% C.I. -8.9 to -0.6; *p* = 0.026), in total light PA at seven months (5.7 mins/day; 95% C.I 1.0 to 10.5; *p* = 0.018), and light PA on school days at 7 months (4.5 mins/day; 0.3 to 8.8; *p* = 0.038). No differences were found in the proportions of participants active at least 60 min over the measurement period at either seven or 14 months.

**Table 5:** Objectively measured activity variables at follow-up between participants randomised to usual practice (control) or to the Girls Active programme (intervention)^a^

	Number of schools | pupils		Adjusted difference at follow-up^b^		
	Control	Intervention	Coefficient (95% C.I.)	*P* value	ICC
MVPA (mins/day)					
School days					
Baseline	10 | 854	10 | 850			
7 months	8 | 602	10 | 746	1.84 (-0.52 to 4.20)	0.126	0.02
14 months	9 | 578	10 | 693	1.79 (-1.27 to 4.85)	0.251	0.02
*Weekend*					
Baseline	10 | 828	10 | 827			
7 months	8 | 561	10 | 702	2.78 (-2.37 to 7.92)	0.290	0.04
14 months	9 | 503	10 | 637	0.99 (-3.54 to 5.51)	0.669	0.02
During school hours					
Baseline	10 | 855	10 | 850			
7 months	8 | 602	10 | 746	0.73 (-0.84 to 2.30)	0.360	0.05
14 months	9 | 578	10 | 693	0.84 (-0.50 to 2.18)	0.218	0.02
After-school hours					
Baseline	10 | 855	10 | 850			
7 months	8 | 602	10 | 746	0.51 (-0.79 to 1.78)	0.443	0.02
14 months	9 | 578	10 | 693	0.39 (-1.61 to 2.39)	0.701	0.03
Average acceleration (ENMO; m*g*/day)					
All days					
Baseline	10 | 858	10 | 850			
7 months	8 | 602	10 | 747	1.39 (0.09 to 2.18)	0.033	0.03
14 months	9 | 578	10 | 694	0.66 (-0.62 to 1.95)	0.314	0.02
School days					
Baseline	10 | 854	10 | 850			
7 months	8 | 602	10 | 747	0.89 (-0.18 to 1.95)	0.102	0.03
14 months	9 | 578	10 | 693	0.46 (-0.91 to 2.02)	0.460	0.03
Weekend					
Baseline	10 | 828	10 | 827			
7 months	8 | 559	10 | 702	1.39 (-1.13 to 3.92)	0.280	0.05
14 months	9 | 503	10 | 637	0.51 (-1.49 to 2.51)	0.619	0.02
Sedentary time (mins/day)					
All days					
Baseline	10 | 848	10 | 846			
7 months	8 | 588	10 | 732	-5.76 (-12.90 to 1.38)	0.114	0.01
14 months	9 | 565	10 | 680	-2.64 (-13.03 to 7.75)	0.618	0.01
School days					
Baseline	10 |840	10 | 844			
7 months	8 | 586	10 | 726	-4.17 (-10.74 to 2.40)	0.213	0.01
14 months	9 | 559	10 | 676	-0.08 (-12.72 to 12.55)	0.990	0.02
Weekends					
Baseline	10 | 815	10 | 806			
7 months	8 | 553	10 | 680	-9.73 (-27.41 to 7.95)	0.281	0.02
14 months	9 | 485	10 | 619	-10.27 (-20.63 to 0.10)	0.052	< 0.001
During school hours					
Baseline	10 | 855	10 | 850			
7 months	8 | 602	10 | 746	3.83 (-4.13 to 11.78)	0.356	0.08
14 months	9 | 578	10 | 693	2.49 (-4.84 to 9.81)	0.506	0.06
After-school hours					
Baseline	10 | 855	10 | 850			
7 months	8 | 602	10 | 746	-4.72 (-8.89 to -0.56)	0.026	0.02
14 months	9 | 578	10 | 693	-1.38 (-6.68 to 3.92)	0.610	0.02
Time spent in light PA (mins/day)					
All days					
Baseline	10 | 858	10 | 850			
7 months	8 | 602	10 | 747	5.71 (0.96 to 10.46)	0.018	0.01
14 months	9 | 578	10 | 694	1.50 (-5.93 to 8.94)	0.692	0.01
School days					
Baseline	10 | 854	10 | 850			
7 months	8 | 602	10 | 746	4.50 (0.25 to 8.75)	0.038	< 0.01
14 months	9 | 578	10 | 694	0.30 (-8.49 to 9.09)	0.947	0.03
Weekends					
Baseline	10 | 828	10 | 827			
7 months	8 | 561	10 | 702	8.85 (-5.52 to 23.22)	0.227	0.05
14 months	9 | 503	10 | 637	3.21 (-6.90 to 13.32)	0.534	0.001
During school hours					
Baseline	10 | 855	10 | 850			
7 months	8 | 602	10 | 746	5.38 (-10.01 to 20.77)	0.493	0.65
14 months	9 | 578	10 | 693	4.08 (-7.55 to 15.72)	0.492	0.54
After-school hours					
Baseline	10 | 855	10 | 850			
7 months	8 | 602	10 | 746	1.70 (-1.30 to 4.69)	0.268	0.01
14 months	9 | 578	10 | 693	0.07 (-4.70 to 4.85)	0.976	0.03
Proportion achieving 60 mins/day of MVPA					
All days					
Baseline	10 | 857	10 | 849			
7 months	8 | 594	10 | 742	0.78 (0.23 to 2.65)	0.688	< 0.001
14 months	9 | 573	10 | 686	0.65 (0.23 to 1.85)	0.420	0.01

*CI* confidence interval; *ICC* intra-class correlation; *PA* physical activity; *MVPA* moderate- to vigorous-intensity physical activity

^a^Based on a complete case analysis

^b^Adjusted difference at follow-up between randomised groups with 95% confidence interval, *P* value, and ICC for schools; adjusted for cluster effect, baseline value, participant year group, school %free school meal eligibility and stratification categories (school size and percentage of non-White pupils)

### Body composition secondary outcomes

No differences between the groups were seen for BMI z-score at either seven (0.003 kg/m^2^; 95% C.I. -0.06 to 0.07; *p* = 0.908) or 14 months (0.02 kg/m^2^; 95% C.I. -0.06 to 0.09; *p* = 0.636). Similarly, no differences between the groups were seen for % body fat at either seven (-0.15%; 95% C.I.-0.83 to 0.52; *p* = 0.656) or 14 months (0.25%; 95% C.I. -0.68 to 1.17; *p* = 0.600).

### Psychosocial secondary outcomes

There was no pattern in the differences found and they were sporadic (Table 6) and slight (questionnaire scales were on a five or seven point scale). At 14 months there was a difference in intentions to being active in favour of the control group (-0.21; 95% C.I. -0.37 to -0.05; *p* = 0.012). At seven months there was a difference in perceived importance in favour of control group (-0.42; 95% C.I. -0.66 to -0.18; *p* < 0.001). At 14 months there was a difference in participants confidence in being active in favour of the control group (-0.08; 95% C.I. -0.14 to -0.02; *p* = 0.013). A significant difference in self-esteem was seen at seven months (0.06; 95% C.I. 0.01 to 0.11; *p* = 0.025) in favour of the intervention group. At 14 months there was a difference between groups perceptions of their school physical environment in favour of the control group (-0.13; 95% C.I. -0.24 to -0.01; *p* = 0.032). At 14 (-0.09; 95% C.I. -0.18 to -0.01; p = 0.032) months there was a difference in identified motivation (motivated by engaging in activities that are a means to an end) in favour of the intervention group.

**Table 6:** Scores for psychosocial measures at follow-up for participants randomised to usual practice (control) or to the Girls Active programme (intervention)

	Number of schools | pupils		Adjusted difference at follow-up^a^		
	Control	Intervention	Coefficient (95% C.I.)	*P* value	ICC
Intentions to be physically active^b^					
Baseline	10 | 871	10 | 842			
7 months	8 | 623	10 | 776	-0.15 (-0.33 to 0.03)	0.094	< 0.001
14 months	9 | 569	10 | 734	-0.21 (-0.37 to -0.05)	0.012	< 0.001
Perceived importance of PA^c^					
Baseline	10 | 849	10 | 828			
7 months	8 | 621	10 | 768	-0.42 (-0.66 to -0.18)	< 0.001	< 0.001
14 months	9 | 557	10 | 729	-0.13 (-0.41 to 0.15)	0.350	< 0.001
Attitudes towards being physically active^d^					
Positive					
Baseline	10 | 877	10 | 863			
7 months	8 | 626	10 | 775	-0.02 (-0.13 to 0.09)	0.775	0.01
14 months	9 | 573	10 | 737	-0.03 (-0.07 to 0.02)	0.250	< 0.001
Negative					
Baseline	10 | 877	10 | 863			
7 months	8 | 627	10 | 775	0.01 (-0.05 to 0.08)	0.661	0.003
14 months	9 | 573	10 | 737	0.04 (-0.04 to 0.11)	0.359	0.001
Whole					
Baseline	10 | 877	10 | 863			
7 months	8 | 627	10 | 775	-0.02 (-0.10 to 0.06)	0.667	0.01
14 months	9 | 573	10 | 737	-0.03 (-0.08 to 0.02)	0.267	< 0.001
Social support for PA from family^e^					
Baseline	10 | 876	10 | 863			
7 months	8 | 626	10 | 774	0.03 (-0.05 to 0.12)	0.445	< 0.001
14 months	9 | 571	10 | 735	-0.05 (-0.11 to 0.01)	0.105	< 0.001
Social support for PA received from peers^e^					
Baseline	10 | 874	10 | 863			
7 months	8 | 626	10 | 773	-0.01 (-0.12 to 0.10)	0.880	0.01
14 months	9 | 567	10 | 734	0.01 (-0.07 to 0.09)	0.762	0.01
School-based social support for PA					
Physical environment^e^					
Baseline	10 | 878	10 | 862			
7 months	8 | 627	10 | 773	0.03 (-0.10 to 0.15)	0.684	0.03
14 months	9 | 571	10 | 734	-0.13 (-0.24 to -0.01)	0.032	0.04
Social environment^e^					
Baseline	10 | 878	10 | 861			
7 months	8 | 626	10 | 773	-0.01 (-0.10 to 0.07)	0.758	0.026
14 months	9 | 570	10 | 734	-0.07 (-0.15 to 0.01)	0.080	0.009
PE teachers^f^					
Baseline	10 | 874	10 | 859			
7 months	8 | 624	10 | 774	-0.03 (-0.23 to 0.16)	0.733	0.026
14 months	9 | 567	10 | 731	-0.21 (-0.45 to 0.02)	0.074	0.018
Confidence to take part in PA (self-efficacy)^d^					
Baseline	10 | 879	10 | 853			
7 months	8 | 625	10 | 774	-0.02 (-0.09 to 0.04)	0.472	< 0.001
14 months	9 | 568	10 | 733	-0.08 (-0.14 to -0.02)	0.013	< 0.001
Enjoyment of PA^g^					
Baseline	10 | 876	10 | 851			
7 months	8 | 625	10 | 772	-0.03 (-0.14 to 0.08)	0.592	0.140
14 months	9 | 566	10 | 731	-0.04 (-0.12 to 0.04)	0.361	< 0.001
Motivation to take part in PA^g^					
Extrinsic					
Baseline	10 | 877	10 | 842			
7 months	8 | 626	10 | 771	-0.02 (-0.11 to 0.07)	0.680	< 0.001
14 months	9 | 568	10 | 735	-0.01 (-0.15 to 0.13)	0.919	0.009
Introjected					
Baseline	10 | 878	10 | 842			
7 months	8 | 626	10 | 771	-0.05 (-0.11 to 0.02)	0.143	< 0.001
14 months	9 | 568	10 | 735	-0.05 (-0.14 to 0.04)	0.260	< 0.001
Identified					
Baseline	10 | 878	10 | 842			
7 months	8 | 626	10 | 771	-0.02 (-0.15 to 0.10)	0.700	0.020
14 months	9 | 568	10 | 735	-0.09 (-0.18 to -0.01)	0.032	< 0.001
Intrinsic					
Baseline	10 | 878	10 | 842			
7 months	8 | 626	10 | 771	-0.01 (-0.18 to 0.18)	0.990	0.028
14 months	9 | 568	10 | 735	-0.06 (-0.16 to -0.04)	0.243	0.002
Amotivation					
Baseline	10 | 877	10 | 841			
7 months	8 | 626	10 | 771	-0.03 (-0.15 to 0.09)	0.578	0.007
14 months	9 | 568	10 | 735	-0.02 (-0.14 to 0.09)	0.684	0.002
Physical self-perception^e^					
Self-esteem					
Baseline	10 | 760	10 | 700			
7 months	8 | 622	10 | 769	0.06 (0.01 to 0.11)	0.025	< 0.001
14 months	9 | 535	10 | 730	-0.06 (-0.13 to 0.01)	0.086	< 0.001
Physical self-worth					
Baseline	10 | 760	10 | 699			
7 months	8 | 622	10 | 769	-0.01 (-0.07 to 0.05)	0.742	0.007
14 months	9 | 535	10 | 730	-0.03 (-0.09 to 0.03)	0.322	0.006
Body attractiveness					
Baseline	10 | 760	10 | 699			
7 months	8 | 622	10 | 769	-0.05 (-0.12 to 0.03)	0.195	< 0.001
14 months	9 | 535	10 | 730	0.02 (-0.05 to 0.08)	0.630	< 0.001

*IQR* interquartile range; *CI* confidence interval; *ICC* intra-class correlation; *PA* physical activity

^a^Based on a complete case analysis, adjusted difference at follow-up between randomised groups with 95% confidence interval, *p* value and ICC for schools; adjusted for cluster effect, baseline value, participant year group, school %free school meal eligibility and stratification categories (school size and percentage of non-White pupils)

^b^Score ranges from 1 = ‘very unlikely’ to 7 = ‘very likely’

^c^Scale ranges from 1 = ‘very unimportant’ to 10 = ‘very important’

^d^Score ranges from 1 = ‘disagree a lot’ to 5 = ‘agree a lot’

^e^Score ranges from 1 = ‘strongly disagree’ to 5 = ‘strongly agree’

^f^Score ranges from 1 = ‘strongly disagree’ to’ 7 = ‘strongly agree’

^g^Score ranges from 1 = ‘no not at all’ to 5 = ‘yes a lot’

### Sensitivity analyses

Sensitivity analyses showed similar results to the main analysis with no differences between groups at 14 months when the various levels of accelerometer data provision were used. The differences found at 7 months in the main analysis were similar when accelerometer data provision was one day or more (2.4 mins/day; 95% C.I 0.13 to 4.62; *p* = 0.038), any 2 days or more (2.4 mins/day; 95% C.I 0.12 to 4.72; *p* = 0.039), and any 3 days or more (2.4 mins/day; 95% C.I 0.02 to 4.7; *p* = 0.048), any 4 or more days (2.3 mins/day; 95% C.I. -0.03 to 4.7; *P* = 0.053) and any three weekdays plus one weekend day or more (2.2 mins/day; 95% C.I. -0.2 to 4.6; *p* = 0.078). The addition of baseline season of data collection to the analysis revealed a difference in MVPA change at 14 month of 0.8 mins/day (95% C.I. 1.2 to 3.5; *p* = 0.027) only. When follow-up season was included in the model there was a difference in MVPA change of 2.8 mins/day at seven months (95% C.I. 0.4 to 5.1; *p* = 0.020).

### Economic analysis

Depending on how Girls Active was implemented costs ranged from an estimated £1054 per year to £3498 per year per school. There were no statistically significant differences found between the groups for health-related quality of life (CHU9D utility index scores) and frequencies and costs of GP and school service use.

### Process evaluation

How each schools implemented the programme was addressed specifically within the process evaluation. In particular, how peer leaders identified and the groups established and run; the positive feelings lead teachers and pupils had about the programme; the challenges and opportunities to implementing a flexible programme within the current educational landscape and school-level constraints to implementation.

## Discussion

Girls Active was designed by the YST to be, and is being delivered as, a flexible programme for UK schools. This paper investigated the effectiveness of the programme in impacting MVPA of adolescent females. PA of this population is of significant public health interest. The complete case, intention-to-treat and per protocol analyses found no evidence of a sustained intervention effect on MVPA between intervention and control groups after 14 months (primary outcome). At the shorter follow-up of seven months, significant differences in MVPA were observed between groups in the complete case, intention-to-treat and per protocol analyses, but the differences in change were small: 2.4, 2.3 and 3.1 mins/day, respectively. When observing MVPA changes in the per-protocol analysis it would seem that implementation of more of the seven core components (as presented in the methods) of Girls Active prevented the MVPA decline i.e. control schools declined by 6.2 mins/day while the intervention schools declined by 1.9 mins/day between baseline and seven months. Due to the flexible nature of the programme lead teachers spent a wide range of time and money on delivering and implementing Girls Active within their schools. The cost-consequence analysis showed no effect on health related quality of life or service use (typical measures utilised in health economic analysis).

The pre-specified subgroups analysis found that the intervention was effective at 14 months in larger schools (+ 3.9 mins/day) but caused an MVPA decrease in smaller schools (-4.4 mins/day). This warrants consideration when designing programmes for roll-out at scale. Although the relative success in larger schools makes intuitive sense, findings from the process evaluation of this study will provide information of the perceived challenges and opportunities in intervention schools. Different strategies within a programme, not necessarily different programmes, may be needed for non-White European girls and late maturing girls based on the findings of the sub-group analysis at seven months.

We saw some differences between groups at seven months for accelerometer variables in favour of the intervention group, but again these differences were relatively small. Little evidence of any changes in potential psychosocial mediators during the course of the programme were found. Process evaluation data will map out timelines of programme activity to help explain these findings.

### Comparison with extant literature

School-based PA interventions targeting adolescents have shown limited success on objective measures of PA, particularly for older adolescents [33]. Recent evidence from school-based trials with a primary outcome of objectively measured MVPA from the UK [34–37], Australia [38, 39] and the Netherlands [40] has emerged. Of these, two were aimed at adolescent girls in secondary schools, [37, 39] and only one reported a significant effect on objective MVPA but in males only [38]. Other studies are currently underway in UK secondary schools which capitalise on influential pupils, mentors, or in-class peer leaders [41, 42].

The findings of this study add to evidence from well-designed, adequately powered trials on the lack of effectiveness of school-based PA programmes on objectively assessed PA [34, 37, 39]. When there is a significant effect, it is ‘small’ (a standardised mean difference ≤ 0.49) [33] highlighting the challenges with intervening on adolescents’ PA within the school setting. Although small effects are seen multi-component programmes and those underpinned by theory may be more effective [9, 13]. Extensive support may be required to show significant but modest intervention effects (e.g. PA4E1 [38]).

This was an evaluation of a programme currently implemented in UK secondary schools so addressing the limitations to the Girls Active programme itself was beyond the scope of this study. Accompanying process evaluation and cost-effectiveness papers will underscore barriers to implementing school-based PA programmes and also demonstrate what resources were actually used to implement the programme, which will be highly valuable for future research and roll-out at scale.

### Strengths and limitations

The strengths of this evaluation study include a fully powered cluster RCT design. The sample was multi-ethnic, incorporating diverse schools. A random sample of 90 girls/schools were included in the evaluation to ensure it was not the “most active” girls who would sign-up. The baseline PA levels would shows that meeting PA levels are low (2.3%) the IQR of MVPA of 29.9–57.0 mins/day would suggest pupils with a wide variety of activity levels were included in this study. Overall, 25.9 to 78.2% of all KS3 girls/school (mean 37.2%) were included making our results generalisable to a whole school population in this age group. However, this may have also acted as a limitation in that over a 14 month period Girls Active is likely to only have had a modest reach, meaning our sample may have not been fully exposed to the intervention. A targeted evaluation sample may have yielded different results. Objective measurement of MVPA as the primary outcome was in line with public heath priorities and meets the need for “objective and comprehensive evaluation” methods for evaluating programmes [43]. Although flexible programme such as Girls Active may benefit from more pragmatic study designs, from a research methods standpoint the RCT design was well received by partners, schools and pupils. Overall, 24% of the schools that were contacted entered the trial (20 out of 82) and schools and pupils were recruited to target. There was good compliance with the accelerometer, low levels of missingness from questionnaires, and participant loss to follow-up was in line with other studies. A full economic analysis was also completed for the Girls Active trial. Based on best-practice, the methods used with schools were designed for this study and the methods used and results found add to the literature on the costs of delivering programmes in the school setting.

## Conclusion

At 14 months, our primary measure of effectiveness was change in MVPA at 14 months. No difference in change in MVPA between Girls Active control and intervention schools was found. At seven months there was less of a decline in MVPA in the intervention schools compared to the control schools. Differences in sub-groups may mean the programme has potential in certain types of schools or pupils. Process evaluation and complete and detailed economic analysis papers will give context and detail of the delivery of such a flexible programme.

## Abbreviations

APHV: Age at peak height velocity
BME: Black and minority ethnicity
BMI: Body mass index
CI: Confidence interval
FSME: Free school meal eligibility
ICC: Intra-class correlation
IMD: Index of multiple deprivation
ITT: Intention to treat
KS3: Key Stage three
LLR: Leicester City, Leicestershire and Rutland
MVPA: Moderate- to vigorous-intensity physical activity
PA: Physical activity
PE: Physical education
RCT: Randomised controlled trial
SD: Standard deviation
SES: Socioeconomic status
UK: United Kingdom
YST: Youth Sport Trust
